# Effectiveness of men in maternity health (MiM) intervention on male involvement in maternal health care to improve maternal health outcomes in Naypyitaw, Myanmar

**DOI:** 10.12688/f1000research.145390.1

**Published:** 2024-08-20

**Authors:** May Chan Oo, San San Myint Aung, Pyae Linn Aung, Alessio Panza

**Affiliations:** 1 College of Public Health Sciences, Chulalongkorn University, Bangkok, Thailand; 2 Myanmar Maternal and Child Welfare Association, Naypyitaw, Myanmar; 3 Myanmar Health Network Organization, Yangon, Myanmar

**Keywords:** Maternal health care, Myanmar, Men in maternity health, Male partner, Institutional delivery

## Abstract

**Background:**

Maternal health care (MHC) is critical for safe motherhood and preventing maternal deaths in Myanmar, but the institutional delivery rates are not yet optimal, increasing preventable maternal deaths. To address this issue, involving men in supporting MHC has been recognized as a strategy to improve MHC outcomes. This study aimed to assess the effectiveness, acceptability, and feasibility of men in maternity health (MiM) intervention on male involvement in MHC and its influence on knowledge, attitudes, and practices related to maternal health and institutional delivery rates in Naypyitaw, Myanmar.

**Methods:**

This study employed a mixed-methods approach with quantitative surveying of the male partners and qualitative interviewing of both male and female partners and health stakeholders. A 6-month MiM education program on pregnancy complications, antenatal care, delivery care, and birth preparedness was provided to the intervention group. Before and after the intervention, comparisons between intervention and control groups were made.

**Results:**

The socio-demographic characteristics of male partners were comparable between the intervention and control groups during the pre-intervention assessment. Before MiM intervention, notable differences in male partners’ knowledge, attitudes, birth preparedness and complication readiness practices regarding MHC were not evident between these two groups. After completing the MiM, significant score improvement, including higher ID rates compared to the control group (p<0.001), was observed. The acceptability and feasibility of the MiM program were contributed by positive responses from qualitative findings, highlighting remarkable changes in the perspectives of male partners in supporting MHC. However, challenges, including financial limitations, cultural influences and a shortage of trained personnel, were encountered.

**Conclusion:**

The MiM program effectively promotes male involvement in MHC, leading to improved MHC outcomes. The MiM intervention offers a promising, evidence-based model to enhance ID rates in Myanmar, requiring tailored approaches to address cultural and financial factors.

## Introduction

The aim of maternal health care (MHC) is to improve the well-being of pregnant women, reduce potential complications, and ensure safe delivery and recovery by means of continuous medical support throughout pregnancy, childbirth, and postnatal phase. ^1^^–^^4^ Enhancing maternal health (MH) is ‘target 3.1: good health and well-being’ of the Sustainable Development Goals and countries employ various strategies to accomplish this target. ^1^

In 2020, approximately 287,000 maternal deaths occurred globally due to causes related to pregnancy and childbirth, which means about 800 women dying daily or one lost every two minutes. Almost 95% of deaths occurred in low and lower-middle-income countries, and the Asia Region accounted for a quarter of them. ^5^ Myanmar, one of the Asia Region, reported 1,700 maternal deaths in 2020. This reflects Myanmar having one of the highest maternal mortality ratios (MMR) at a rate of 179 per 100,000 births compared to the global MMR of 223 per 100,000 live births. ^6^ Despite ongoing efforts, most of these preventable deaths persist, especially in resource-limited settings. ^6^^–^^8^

In recent years, Myanmar has made efforts towards improving the utilization of MHC services, but there are still gaps that need addressing. According to the 2015-2016 national demographic survey, 95% of pregnant women received antenatal care (ANC) by skilled health care providers at least once, but 63% received the recommended minimum of four ANC visits. Furthermore, 37% had access to skilled birth attendants in health facilities known as institutional delivery (ID), which is attributed to inadequate health care facilities, transportation difficulties and geographical barriers. In Myanmar, there are still disparities in accessing ID services, with 39% of women in rural areas and 77% of women in urban areas using health facilities for childbirth. This highlights the need for actions to improve access in regions that have limited resources. ^9^^,^^10^

The 1994 Cairo International Conference on Population and Development (ICPD) emphasized the importance of male involvement (MI) in reproductive health and gender equality. ^11^ In 2015, the recommendation from WHO emphasized the importance of men supporting women’s well-being throughout the stages of pregnancy, childbirth and postpartum. ^12^ This definition, which is endorsed by UNFPA ^13^ and used in this manuscript, considers both “males” and “men” as terms. Over time, studies have consistently shown that involving men in MH initiatives leads to improved MHC, increased awareness about MH issues, and better communication and support among couples, all of which benefit families and communities. ^14^^–^^18^

While the previous 2011-2016 five-year national health plan overlooked MI in MH, there has been a notable shift in the 2017-2021 plan by recognizing the role of men in promoting MH and advancing gender equality in Myanmar. The Ministry of Health in Myanmar has identified MI as a strategy for improving MH outcomes, and it has been explicitly incorporated into the national health policy. ^19^

Despite extensive searches on Medscape, PubMed, Google Scholar, and ProQuest with keywords “male/men/husband involvement” “maternal health care” “Myanmar”, limited research explores MI in MHC in Myanmar. Existing studies have mainly relied on quantitative, observational, community-based approaches to describe men’s knowledge and perceptions of pregnant women and their involvement in MHC practices. ^20^^–^^22^ Moreover, these studies carried out only the quantitative approaches without incorporating mixed-method designs or interventions for exploring ID. Therefore, the objective of this study is to explore the effectiveness, acceptability, and feasibility of the men in maternity health (MiM) intervention on MI in MHC, including its impact on knowledge about MH-related issues, attitudes towards MHC and birth preparedness and complication readiness (BPCR) practices and improving ID rates through a mixed-method approach. Achieving this objective in the patriarchal context of Myanmar would be highly valuable to provide an evidence-based model for increasing ID and improving MH outcomes.

## Methods

### Study design and setting

A quantitative-qualitative sequential, explanatory mixed-methods study was conducted. The quantitative methods involved pre- and post-interventional surveys, while the qualitative methods included focus group discussions (FGDs) with MH service users and in-depth interviews (IDIs) with manager-level and field-level MH service providers.

This study was implemented in Naypyitaw territory, where ID was 35.8% in 2014. ^9^ Naypyitaw encompasses two districts, each comprising four townships. Lewe Township from Dekkhina district was purposively selected for its high population density and middle socio-economic status as the intervention group. Takkone Township from Ottara district, with a comparable population density and socioeconomic status, served as the control group.

### Quantitative research

**Study population, sample size and participants selection**

This study included men aged 18 years or older with primigravida female partners who were at least 16 weeks pregnant at the time of the intervention, residing in the study areas, and providing consent. In each intervention and control township, five health centres serving a high number of pregnant women were purposively selected. Midwives from these centres provided a list of eligible men whose female partners were pregnant. Using systematic random sampling, a total of 198 men (99 in each group) were selected to achieve the required sample size. Sample size calculations followed the two independent proportion formula, adapted from a study in Ethiopia, a low-income country comparable to Myanmar in terms of MMR and MHC provision. ^23^

**Men in Maternity Health (MiM) Intervention**

The six-month MiM education program focused on MH education for male partners of pregnant women in the intervention area. Topics included pregnancy and its complications, obstetric danger signs, the importance of MHC, including ANC, delivery care, and postnatal care, the benefits of BPCR, and safe delivery. Invitation letters were sent to male partners by public health supervisors at the health centres every first and third week to encourage participation in the MiM program’s activities. Two-hour-long education and discussion sessions occurred in five selected health centres every second and fourth week on Sundays, respectively, considering participants’ work schedules. Assigned midwives offered face-to-face health education using flip charts, distributed pamphlets, and organized group discussions, following the guidelines in the “Standardized Health Messages Handbook”. ^5^ During discussions, male partners were encouraged to share their experiences, knowledge, and intentions regarding the adoption of new MHC-related behaviours, including topics covered in education sessions. Prior to implementation, providers were trained to deliver the MiM program’s education curriculum and guidelines. Attendance consistently remained high at 80% of participants every month, with home visits for absentees to provide health education.

### Data collection

A quantitative survey, employing structured questionnaires, was conducted in a private room at the health centre both before and after the intervention and childbirth. The pre-intervention assessment took place from December 2018 to January 2019, and the post-intervention assessment from July to August 2019. The questionnaire was initially prepared in English, adapting Johns Hopkins Program for International Education in Gynecology and Obstetrics (JHPIEGO)’s monitoring BPCR tools, ^24^ which had been tested for validity in low-income settings similar to Myanmar. The questionnaire was then translated into Burmese and back-translated into English. Ten village health volunteers, all holding bachelor’s degrees, were trained as interviewers for data collection. The researcher validated reported ID by cross-referencing birth certificate records and obtaining information from assigned area midwives. Quantitative data collection lasted approximately 45 minutes. Pilot testing of questionnaires was conducted in a socio-economically and geographically similar township, strategically distant to avoid cross-contamination with study townships.

### Quantitative data analysis

The Excel database developed by the researcher was used for quantitative data entry. Descriptive statistics, including frequency, percentage, mean, standard deviation, minimum, and maximum, were used for numerical variables. For categorical variables, frequency and percentage were employed. To evaluate group homogeneity, inferential statistics included paired t-tests for within-group analysis, independent t-tests for between-group comparisons, and chi-square tests for categorical data. For the practices of MI in BPCR, five key elements: preparing the health facility for birth, ensuring the presence of skilled birth attendants, arranging transportation for delivery or emergencies, identifying potential blood donors and saving money for delivery expenses, were considered. The scores ranged from 1 (‘Yes’) to 0 (‘No’) on a scale of 0 to 5. The Statistical Package for the Social Sciences (SPSS) version 28 (IBM Corp., Armonk, NY, USA) was used for analyzing the quantitative data.

### Qualitative research

The FGDs and IDIs were conducted in the intervention township to gain a comprehensive understanding of the impact of the MiM program, exploring how acceptable and feasible the program is for preventing maternal emergencies and promoting IDs.

To ensure privacy and confidentiality, FGDs were organized in private rooms. Employing extreme case sampling, couples were chosen based on MI scores in BPCR practices related to maternal emergencies and their female partners’ ID status. Similarly, couples were selected where male partners had the lowest MI scores and their partners did not possess IDs. A total of eight FGDs were conducted separately; four sessions for male partners and four for their female partners, aiming to recruit 6-8 participants for each session.

Simultaneously, IDIs were carried out with participants involved in the MiM program, including MH program managers (Township Medical Officers and Health Assistants) overseeing its implementation and MH providers (Lady Health Visitors, or Midwives). Participants included in IDIs were purposively chosen based on their roles in the MiM program, resulting in a total of 11 MH program stakeholders from the intervention township. Pilot testing of IDI and FGD guidelines was also conducted within the same township as the quantitative pilot testing. On average, IDIs and FGDs lasted around 60 and 90 minutes, respectively.

### Qualitative data analysis

For the qualitative component, IDIs and FGDs were audio-recorded, transcribed verbatim, and analyzed thematically using the framework method. This analysis employed a combination of inductive and deductive techniques. Descriptive and analytical coding was applied to the transcripts to identify overarching themes and sub-themes, which are presented in the study findings. Qualitative data analysis were performed using NVivo version 12.

### Ethics approval and consent to participate

This study protocol was approved by the Ethics Review Committee on Medical Research Involving Human Subjects, Department of Medical Research, Ministry of Health, Yangon, Myanmar (Approval No: ERC/DMR/2018/133) on 5 ^th^ October 2018. Before data collection, participants provided written informed consent, with confidentiality maintained throughout all stages of data collection, management, analysis, and reporting. This study was retrospectively registered with ClinicalTrials.gov on June 11, 2024 (NCT06451653), due to an initial oversight regarding the necessity of prior registration, which was corrected upon recognizing of its importance for transparency and adherence to guidelines.

The study was conducted in Naypyitaw, Myanmar, with most of the authors being Myanmar nationals. According to Myanmar’s regulations, research conducted within the country should receive ethical approval from an authorized local IRB. Consequently, we obtained approval from the Ethics Review Committee on Medical Research Involving Human Subjects, Department of Medical Research, Ministry of Health, Yangon, Myanmar. This committee, like the IRBs at the authors’ affiliated institutions, adheres to the Declaration of Helsinki. Choosing a local IRB ensured compliance with local ethical standards, streamlined the approval process, and ensured adherence to local regulations and guidelines. Additionally, while the first author is an academic researcher at Chulalongkorn University, the study’s location and context necessitated adherence Myanmar’s ethical procedures.

## Results

### Socio-demographic characteristics of the study participants in intervention and control groups

Participants’ socio-demographic characteristics were equated for valid comparison of the intervention’s effects between the intervention and control groups. In the intervention group, male partners’ mean age was 29.64±5.68 years, while in the control group, it was 30.44±6.09 years. Both groups had similar educational levels, manual occupations, monogamous marriages, and living arrangements. While assessing economic status using the national quintile of wealth index, the majority of participants were in the highest (39.4%) and fourth (39.9%) levels. No significant differences were found between the groups (p>0.05), ensuring the validity of comparing the intervention effects at both time points ( Table 1).

**Table 1.** Socio-demographic characteristics of male partners in intervention and control group.

Characteristics	Study group (n=99)	Control group (n=99)	Total (n=198)	^a^ p-value
	n (%)	n (%)	n (%)	
**Age of male partners (years)**				
18-29	54 (54.5)	45 (45.5)	99 (50.0)	0.381
30-39	40 (40.4)	46 (46.5)	86 (43.4)	
≥40	5 (5.1)	8 (8.0)	13 (6.6)	
	29.64 ± 5.68	30.44 ± 6.09		
**Education of male partner**				
Illiterate or no formal education	4 (4.0)	6 (6.1)	10 (5.0)	0.969
Primary education level	28 (28.3)	29 (29.3)	57 (28.8)	
Middle education level	34 (34.3)	32 (32.3)	66 (33.3)	
High education level	25 (25.3)	25 (25.3)	50 (25.3)	
University/College education level	8 (8.1)	7 (7.0)	15 (7.6)	
**Occupation of male partners**				
Self-employee	37 (37.4)	41 (41.4)	78 (39.4)	0.776
Manual worker	54 (54.5)	49 (49.5)	103 (52.0)	
Others	8 (8.1)	9 (9.1)	17 (8.6)	
**Type of marriage**				
Monogamous	92 (92.9)	95 (96.0)	187 (94.4)	0.352
Polygamous	7 (7.1)	4 (4.0)	11 (5.6)	
**Live with mother/mother-in-law**				
No	59 (59.6)	67 (67.7)	126 (63.6)	0.237
Yes	40 (40.4)	32 (32.3)	72 (36.4)	
**Wealth index**				
Highest	36 (36.4)	42 (42.4)	78 (39.4)	0.463
Fourth	38 (38.4)	41 (41.4)	79 (39.9)	
Middle	13 (13.1)	8 (8.1)	21 (10.6)	
Second	12 (12.1)	8 (8.1)	20 (10.1)	
Lowest	0 (0.0)	0 (0.0)	0 (0.0)	

^a^ p-value by Pearson’s Chi-square test; Degree of freedom for Chi-square test=1.

### Effect of MiM intervention on maternal health knowledge and attitude among the intervention and control groups before and after intervention

The impact of MiM intervention on knowledge and attitudes was evaluated by mean scores comparison between intervention and control groups ( Table 2). Before the intervention, there was no difference in MH knowledge between the groups (p=0.987). However, after the intervention, a notable difference emerged (p=0.001), with the intervention group scoring (39.2±10.9) compared to the control group (31.4±13.6). Similarly, attitude scores showed no statistically significant pre-interventional difference (p=0.664) but a significant post-intervention difference (p=0.044). The intervention group presented higher attitude scores (38.4±4.7) at post-intervention, while the control group’s scores remained consistent (36.5±4.0). Within-group analyses demonstrated significant improvements in knowledge and attitudes towards MH following the MiM in the intervention group (p<0.001). Additionally, the control group also experienced a significant increase in knowledge scores post-intervention compared to pre-intervention assessment (p<0.001).

**Table 2.** Mean scores and mean difference of knowledge and attitude among two groups before and after intervention.

Description	Pre Intervention				Post Intervention				Pre/Post			
	Study	Control	Mean difference (95% CI)	p-valueª	Study	Control	Mean difference (95% CI)	p-valueª	Study		Control	
									Mean difference (95% CI)	p-value ^b^	Mean difference (95% CI)	p-value ^b^
**Total knowledge (mean ± SD)**	28.8±12.9	25.5±12.4	3.3 (-0.2,6.8)	0.987	39.2±10.9	31.4±13.6	7.9 (4.4,11.3)	0.001 *	10.4 (7.4, 13.5)	<0.001 **	5.9 (3.3, 8.4)	<0.001 **
ANC	11.4±3.1	11.1±2.7	0.3 (-0.5,1.1)	0.067	14.1±2.1	12.8±3.7	1.3 (0.4,2.1)	<0.001 **	2.7 (1.9, 3.4)	<0.001 **	1.7 (0.9, 2.6)	<0.001 **
Pregnancy danger signs	5.6±3.9	4.3±3.9	1.3 (0.2, 2.4)	0.127	7.5±3.8	5.4±4.3	2.1 (0.9, 3.2)	0.006 *	1.9 (0.8, 2.9)	<0.001 **	1.1 (0.2, 1.9)	0.013 *
Delivery danger signs	4.7±3.2	4.2±3.2	0.5 (-0.4,1.4)	0.820	6.6±2.8	4.7±3.4	1.8 (0.9,2.7)	0.003 *	1.9 (1.1, 2.6)	<0.001 **	0.5 (-0.2, 1.2)	0.176
Postpartum danger signs	3.1±2.6	2.5±2.5	0.6 (-0.9, 1.3)	0.779	4.8±2.7	3.7±2.8	1.1 (0.4, 1.9)	0.262	1.7 (0.9, 2.5)	<0.001 **	1.2 (0.7, 1.8)	<0.001 **
Maternal health care	4.0±3.2	3.4±3.2	0.6 (-0.3, 1.5)	0.329	6.3±2.5	4.8±3.3	1.5 (0.7, 2.4)	<0.001 **	2.3 (1.6, 3.1)	<0.001 **	1.4 (0.6, 2.2)	0.001 *
**Total attitude (mean±SD)**	36.2±3.7	35.9±3.6	0.2 (-0.8, 1.2)	0.664	38.4±4.7	36.5±4.0	1.9 (0.7, 3.1)	0.044 *	2.3 (1.2, 3.4)	<0.001 **	0.5 (-0.3, 1.4)	0.215

p-valueª – between group comparison using independent t-test, p-value ^b^ - within group comparison using paired t-test,

* Significance at p-value<0.05.

** Significance at p-value<0.001.

The IDI interviews with health providers revealed that male partners initially lacked awareness regarding their role in supporting pregnancy and childbirth. However, through the influence of MiM program activities, male partners’ knowledge, attitudes, and support improved significantly, leading to increased utilization of MH services—particularly through active involvement during ANC visits.

*“Prior to implementing MiM, many women faced lack of support from their male partners when accessing maternal health services. The MiM encouraged male partners to accompany their wives during ANC visits, and this enhanced the utilization of maternal care services in health facilities.”* (Manager-level health provider, IDI)

Moreover, the FGD revealed a change in the attitude of male partners towards their roles in pregnancy and childbirth.

*“Prior to participating in MiM, my husband had limited awareness of his role in pregnancy and childbirth beyond providing financial support. I also held the same perspective.”* (Female partner, female FGD)

*“I experienced swelling in my legs and high blood pressure when pregnant, he accompanied me to ANC visits, reminded me about danger signs, prepared for emergencies, and even helped with household chores that he had never done before.”* (Female partner, female FGD)

### Effect of MiM intervention on birth preparedness and complication readiness (BPCR) practices among the intervention and control groups before and after intervention

The study assessed the effects of 6-month MiM intervention on MI in five key BPCR practices. Before the intervention, there were no significant differences between the two groups. However, after the intervention, a significant difference was observed between the intervention and control groups (p<0.05). The within group analysis revealed an increase in BPCR practices among participants receiving the MiM intervention (p<0.05). Male partners in the control group were more likely to secure skilled birth attendants during post-assessment, compared to pre-intervention assessment (p=0.019) ( Table 3).

**Table 3.** Effect of MiM intervention on male involvement (MI) in BPCR before and after intervention among study and control group.

Characteristics	Study group (n=99) n (%)	Control group (n=99) n (%)	p-valueª
MI in BPCR:			
a) MI in planning for delivery place			
Before intervention	60 (60.6)	62 (62.6)	0.770
After intervention	92 (92.9)	68 (68.7)	<0.001 **
p-value ^b^	<0.001 **	0.369	
b) MI in arranging skill birth attendant			
Before intervention	56 (56.6)	53 (53.5)	0.668
After intervention	88 (88.9)	69 (69.7)	0.001 *
p-value ^b^	<0.001 **	0.019 *	
c) MI in saving money			
Before intervention	78 (78.8)	80 (80.8)	0.723
After intervention	93 (93.9)	81 (81.8)	0.009 *
p-value ^b^	0.002 *	0.855	
d) MI in planning for transportation			
Before intervention	54 (54.5)	59 (59.6)	0.473
After intervention	81 (81.8)	61 (61.6)	0.002 *
p-value ^b^	<0.001 **	0.771	
e) MI in planning for blood donor			
Before intervention	34 (34.3)	33 (33.3)	0.881
After intervention	66 (66.7)	36 (36.4)	<0.001 *
p-value ^b^	<0.001 **	0.655	

p-value ^a^ - between group comparison using Pearson’s Chi square test, p-value ^b^ - within groups comparison using Pearson’s Chi square test.

* Significance at p-value<0.05.

** Significance at p-value<0.001.

Both managers and field-level health providers recognized that a higher proportion of couples in the intervention group made childbirth plans according to BPCR guidelines, leading to safer deliveries and fewer maternal emergencies. During FGDs with male partners, it was evident that they actively participated in ensuring safe childbirth; however, their level of engagement varied depending on factors such as occupation, family size, and risk perceptions.

*“My occupation as a manual worker, living with my parents, and insufficient daily wages due to my large-sized family challenged me to save money for delivery and related costs.”* (Male partner, male FGD)

*“I didn’t arrange a potential blood donor while my wife was pregnant because I didn’t expect any health issues to arise, given her overall good health throughout the pregnancy.”* (Male partner, male FGD)

### Effect of MiM intervention on institutional delivery (ID)

The study assessed the impact of MiM on ID rates, revealing that intervention group had a higher prevalence (64.6%) compared to the control group (39.4%), and this difference was statistically significant (p<0.001) ( Table 4).

**Table 4.** Prevalence of Institutional delivery among study and control group after intervention.

Characteristics	Study group (n=99)		Control group (n=99)		p-valueª
	n	%	n	%	
Institutional delivery	64	64.6	39	39.4	<0.001 **
Home delivery	35	35.4	60	60.6	

p-value ^a^ - between group comparison using Pearson’s Chi square test,

** Significance at p-value<0.001.

A manager-level health provider reported that a considerable proportion of women in the intervention group from rural areas favored home deliveries despite increased MH service utilization.

*“Although there was an increase in ANC utilization and a doubling of institutional delivery rates in the intervention township, about one-third of rural women still opt for home delivery due to the distance from health facilities.”* (Manager-level health provider, IDI)

While most women had experiences with their male partners in BPCR activities, some encountered challenges such as cultural influences that favored home delivery and financial constraints.

*“I chose home delivery with my mother and a traditional birth attendant, so it eliminated the need for my husband to arrange transportation to a delivery facility.”* (Female partner, female FGD)

*“Despite my husband’s advice for hospital delivery, I chose home delivery due to strong encouragement from my mother and aunt, who had personal experience with it.”* (Female partner, female FGD)

### Acceptability of the MiM program

The acceptability and feasibility of the MiM program were assessed exclusively through qualitative interviews. Health providers highlighted the positive response from participants towards the MiM as male partners expressed an understanding of MH and recognized their role in ensuring safe delivery.

*“At first, some men resisted attending the sessions because they felt that maternal health was not their responsibility and were unfamiliar with the program. However, educational efforts helped them realize their importance leading to increased attendance in the MiM program over time.”* (Field-level health provider, IDI)

FGDs with male partners revealed that they gained more confidence in supporting their female partners during pregnancy and became more willing to engage in discussions. Women preferred the program due to its positive impact on their partners’ knowledge and behavior, particularly including healthy pregnancy outcomes through nutrition.

*“I first hesitated to ask questions but later on gained confidence and took part in the activities as facilitators created an enabling environment and assured us (male partners) that no question was basic or silly during the sessions.”* (Male partner, male FGD)

*“After the MiM program, my husband learned about the necessity of balanced nutrition during pregnancy. Consequently, he ensured my balanced diet and regular intake of iron and folic acid supplements, which led to the birth of our baby without any complications.”* (Female partner, female FGD)

The program enabled male partners to engage with MHC providers, addressing concerns and providing tailored materials for specific MH needs.

*“Male partners gained new skills in birth preparedness, including knowledge about arranging blood donors in case of emergency situations. In our community, where health literacy is low, many people are unaware of their blood group.”* (Field-level health provider, IDI)

Both men and their partners recognized the impact of the program on their roles and improved MH outcomes by promoting the identification of issues and preventing complications.

*“When I experienced vaginal bleeding, my husband arranged me to go for regular check-ups and accompanied me to the hospital. It was revealed that the placenta had implanted at the bottom of my uterus. Because of his support, the condition was detected early, and I received timely medical intervention to avoid a potential emergency.”* (Female partner, female FGD)

### Feasibility of the introduced MiM

**Technical feasibility**

Manager-level health providers emphasized the feasibility of MiM in terms of its technical aspects, specifically mentioning its design and ease of comprehension for male partners who have limited literacy skills.

*“MiM’s educational tools are designed for easy understanding by the intended audience, especially those with limited literacy or health knowledge. The tools are user-friendly for facilitators during MiM sessions, aligning well with community standards. The use of visual aids addresses the challenges and concerns that men face during their wives’ pregnancies, providing valuable support tailored to meet the needs of the community.”* (Manager-level health provider, IDI)

Men and their partners found the health education materials, particularly the visuals, easily comprehensible, effectively conveying essential information on MH and emergency preparedness.

*“Although I can’t read as proficiently as others, I can still understand the information by looking at the visual aids and listening to the health education provided by the midwife.”* (Male partner, male FGD)

*“I observe the pictures in the books and pamphlets provided by the MiM to explain the necessary actions related to maternal health and preparedness for emergencies to my husband. This also makes it easier for me to grasp what needs to be done.”* (Female partner, female FGD)

**Time feasibility**

Regarding the schedule of MiM, health providers emphasized the need for flexibility to accommodate the time constraints of male partners. Additionally, most men expressed their preference for weekend sessions and convenient locations, while some suggested shorter and more focused session durations to enhance engagement.

*“Some sessions were long, which made it difficult for us* (male partners) *to maintain focus and engagement throughout. If these sessions were divided into shorter and more manageable parts, it could improve our overall learning experience.”* (Male partner, male FGD)

IDIs with health providers recommended a suitable program duration of six months for the male partners to acquire new skills. In FGDs, some men found that a six-month program duration was adequate for learning while others suggested it should be shorter due to their early knowledge acquisition.

*“I think six-month program is too long. I gained the knowledge and skills needed within the first few months. With the varied needs of different individuals, some taking longer times, a shorter program for others within this duration should be considered.”* (Male partner, male FGD)

**Operational feasibility**

Manager-level health providers addressed the challenges by engaging with the community and closely monitoring the process of the program. Field-level providers acknowledged that the implementation of MiM program has shown feasibility and positive outcomes. However, both emphasized the need for additional trained personnel with skills in engaging male partners to ensure the successful implementation of MiM.

*“The MiM yielded positive outcomes with an increase in institutional deliveries and maternal health practices. However, for program sustainability, trained personnel are needed to consider as we have our existing duties and limited staff resources.”* (Manager-level health provider, IDI)

**Economic feasibility**

Both manager- and field-level providers highlighted that one of the advantages of MiM is its feasibility, as it is offered free of charge, which encourages active participation. During FGDs with male partners, it was observed that they expressed favorable views regarding affordability but mentioned concerns about transportation costs and potential loss of daily income for those engaged in manual labor. Similarly, women acknowledged its affordability; however, one participant expressed concerns about her husband’s work absence during MiM sessions.

*“Transportation costs are required to attend the MiM sessions. Unlike others, as a manual worker, I lost my daily income while participating in the MiM education sessions.”* (Male partner, male FGD)

*“As my husband is the breadwinner in our family, he faced work challenges due to attending MiM education sessions since we depend on his income. Fortunately, we were able to resolve this by consulting with his employer, who let him attend during his break time and make up for the work he missed at another time.”* (Female partner, female FGD)

## Discussion

This study was carried out as part of the Maternal and Reproductive Health program in Myanmar to explore the effectiveness of MiM programme on MI with regard to improving MHC and its impact on ID. This study employed a mixed-methods approach, including quantitative surveys and qualitative interviews, and ultimately found significant increases in knowledge, attitudes, and BPCR practices among male partners in the intervention compared to the control group.

The MiM program aimed to address gaps in MH in Myanmar, where ID rates are not optimal. By providing targeted education and facilitating interactive discussions, MiM effectively addressed the initial lack of knowledge and limited awareness among male partners about their role in supporting pregnancy and childbirth. The intervention significantly changed the male partners’ attitudes towards their involvement in pregnancy and childbirth. These findings are consistent with evidence from an interventional quasi-experimental study conducted in Tanzania that involved men with pregnant partners, as well as another study in Nigeria that employed a stepped-wedge cluster randomized controlled trial involving spouses of pregnant women. Both studies showed improvements in knowledge and attitudes among male partners regarding MH following the intervention. ^25^^,^^26^ Interestingly, in this study, the post-intervention knowledge score unexpectedly increased in the control group due to MH education received from various sources, including neighbors, local MHC providers, and mass media. Since 2014, MH education programs have been disseminated through radio and TV series, as well as mobile applications like ‘May May’ and ‘Phay Phay’. ^27^

According to the White Ribbon Alliance and JHPIEGO, it is more effective to prevent maternal emergencies rather than just managing them after they occur. It is crucial to adhere to BPCR practices to avoid negative consequences, particularly concerning birth plans. ^24^^,^^28^ In this study, the MiM intervention significantly improved BPCR practices among male partners. In contrast, a quasi-experimental intervention study involving married men in Nigeria presented low MI in BPCR practice possibly due to religious misconceptions. ^29^ However, a randomized controlled intervention study conducted in Gambia, focusing on spouses of pregnant women, reported an increase in BPCR practices, ^30^ which aligns with the findings of the MiM intervention. These findings highlight the importance of targeted interventions like the MiM program in improving MI in MHC and ultimately contributing to better MH outcomes. Furthermore, in this study, male partners in the control group showed an increase in planning for skilled birth attendants during the post-assessment, possibly because even when their wives chose home delivery, they needed to arrange for a traditional birth attendant.

Previous randomized controlled intervention studies conducted in Nepal and Gambia have highlighted the impact of involving male partners in MHC. These studies have shown that when men actively support their wives during pregnancy and childbirth, it leads to improved MH outcomes and increased utilization of MH utilization. ^30^^,^^31^ This MiM study supports those findings by demonstrating an increase in ID among intervention group compared to the control group. The intervention effectively empowered male partners to play an active role, which positively influenced women’s decisions to opt for ID. However, despite the success of the intervention, a considerable number of women in both groups still choose home deliveries. This suggests that cultural influences and financial constraints continue to impact the uptake of ID services. These findings are consistent with qualitative research conducted in Ghana and Uganda that also emphasized how sociocultural norms and practices influence delivery decisions. This underscores the need for targeted interventions aimed at promoting IDs. ^32^^,^^33^

The qualitative data provided insights into the acceptability and feasibility of MiM program. Both male and female participants expressed responses emphasizing its effectiveness in improving learning, communication, and MH outcomes. The assessment of feasibility demonstrated that the program is technically and operationally viable with participants giving feedback on its design of visual aids and accessibility of health education materials. However, health stakeholders highlighted the need for trained personnel due to challenges in human resources. These findings emphasize the importance of providing training and support to health care providers for the successful implementation of the program, aligning with realist reviews and discussion studies on MI in low- and middle-income countries. ^34^^,^^35^ Flexible scheduling played a role in accommodating time constraints and competing demands for male partners thereby enhancing operational feasibility. While participants viewed the feasibility of MiM positively since it is a free program, concerns were raised about transportation costs and income loss for male partners. This highlights the importance of addressing financial constraints to enhance MI, which aligns with similar findings, from mixed-method studies conducted in Kenya and a qualitative study in Malawi. ^36^^,^^37^

Overall, this present study provides important new insights into how and why the MiM intervention is useful in promoting MI in MH and increasing ID rates in Myanmar, thereby showing evidence-based programs to improve MH outcomes. The results show that partner-targeted interventions can be effective with a positive change in terms of knowledge, attitudes, and practices regarding MHC. Similarly, planned interventions targeting other areas and populations of the country could be helpful in advancing MH outcomes. However, challenges such as cultural influences, financial constraints as well as trained personnel need to feature in future implementations so that the intervention has an optimized effect. The effectiveness of the MiM program may vary across diverse settings, requiring suitable approaches to address particular MI challenges that exist in MHC setting.

### Strength and limitation of the study

This study employs a quantitative-qualitative sequential explanatory mixed-methods approach with methodological rigor, integrating pre- and post-intervention surveys with IDIs and FGDs. It represent the first intervention study with male partners to improve knowledge, attitudes, and BPCR practices for safe motherhood and ID in Nay Pyi Taw. The MiM intervention’s tailored focus on male partners addresses community-specific needs, resulting in increased ID rates through health education and discussion. Additionally, the use of systematic random sampling ensures the reduction of selection bias, while comprehensive training for data collectors is designed to minimize bias. These strengths enhance the credibility and validity of the findings, providing valuable insights into the MiM’s effectiveness in improving MH outcomes.

Despite its strengths, this study was conducted in two purposively chosen townships in Nay Pyi Taw Union Territory, thus limiting generalizability within Myanmar or beyond. The six-month intervention may have limited the assessment of long-term MiM impact, and this would justify a longer follow-up to understand changes over time. External co-interventions, such as mass media and information from nearby health providers, could influence the outcomes of the study. About 20% of the participants missed monthly MiM activities at health centres, which hampered collective learning through group discussions and overall intervention effectiveness, despite efforts to provide MiM face-to-face education at home. Future MI-focused interventions in MHC should, therefore, address such limitations for stronger impact and applicability in similar settings.

## Conclusions and recommendation

This study provides compelling evidence that MiM program markedly improved male partners’ knowledge, attitudes, and practices concerning MH in Myanmar, leading to a noticeable increase in ID prevalence. The findings highlight the critical role of men in MHC in creating an enabling environment for safe deliveries and improved MH outcomes. Continuation of the health education sessions through well-trained field-level health staff is strongly recommended, subject to receiving adequate support for specific MI guidelines and budget for training health staff from the central-level Ministry of Health. It is also suggested to establish community support groups involving male and female members as interactive platforms to strengthen MI’s advantages in MHC. This would extend such initiatives to different settings and populations, contributing to a broader MH promotion. Lastly, further research, including longitudinal studies to reveal insights into MiM program’s long-term impact, along with qualitative studies focusing on male and female points of view with health care providers, is recommended to reveal barriers and facilitators towards continued MI in MHC and to refine interventions for promoting MI in the future.

## Data Availability

Qualitative data cannot be made publicly available according to the Ethics Review Committee on Medical Research Involving Human Subjects, Department of Medical Research, Ministry of Health, Yangon, Myanmar because the datasets generated and/or analysed during the current study comprise collected data from specific districts and townships in Naypyitaw, Myanmar, and the information may be identifiable to particular individuals, risking a breach in confidentiality. Qualitative data (limited de-identified transcripts of interviews) can be requested by contacting to the first author, May Chan Oo ( maechanoo@gmail.com) under no strict conditions. Please note that access to these transcripts is subject to limitations related to participant privacy and data sensitivity, and are available only in Burmese language. Figshare: Effectiveness of men in maternity health (MiM) intervention on male involvement in maternal health care to improve maternal health outcomes in Naypyitaw, Myanmar. https://doi.org/10.6084/m9.figshare.25039013. This project contains the following underlying data:
- Quantitative data Figshare: Supplementary materials for the “effectiveness of men in maternity health (MiM) intervention on male involvement in maternal health care to improve maternal health outcomes in Naypyitaw, Myanmar” are available in https://doi.org/10.6084/m9.figshare.25039013. This project contains the following extended data:
- Information sheet and informed consent forms - Survey questionnaire - Interview and focus group discussion guides Figshare: COREQ and TREND checklists for Effectiveness of men in maternity health (MiM) intervention on male involvement in maternal health care to improve maternal health outcomes in Naypyitaw, Myanmar. in https://doi.org/10.6084/m9.figshare.25039013. All data are available under the terms of the Creative Commons Attribution 4.0 International license (CC-BY 4.0).
