# Supplementary material for: Effectiveness of men in maternity health (MiM) intervention on male involvement in maternal health care to improve maternal health outcomes in Naypyitaw, Myanmar
Source: F1000Res. 2024 Aug 20;13:939. [Version 1] doi: 10.12688/f1000research.145390.1 (PMC11803201; doi:10.12688/f1000research.145390.1)
Supplement: Peer review history

# Reviewer report: Reviewer response for version 1

**DOI:** 10.5256/f1000research.159331.r350079

**Published:** 2025-01-06

**Authors:** Lafi Munira

**Affiliations:** 1 National Research and Innovation Agency, Jakarta, Indonesia

1) Introduction: Could you please include similar intervention studies on men's involvement from other countries or neighboring regions with similar patriarchal cultures, if available?

 2) Method: Please add 1–2 sentences explaining the reasons for choosing a sequential explanatory mixed-methods design, supported by references.

 For example: 'This design was chosen to first quantitatively examine the phenomena of interest and subsequently provide in-depth qualitative insights to contextualize and explain the quantitative findings (Creswell & Plano Clark, 2018).

 3) Method - Measurement Tools:For the quantitative section, could you please elaborate on the measurement tools used, including a detailed description of the dependent and independent variables? Additionally, specify the cut-off points for each measurement where applicable.

Is the work clearly and accurately presented and does it cite the current literature?

Yes

If applicable, is the statistical analysis and its interpretation appropriate?

Yes

Are all the source data underlying the results available to ensure full reproducibility?

Yes

Is the study design appropriate and is the work technically sound?

Yes

Are the conclusions drawn adequately supported by the results?

Yes

Are sufficient details of methods and analysis provided to allow replication by others?

Yes

Reviewer Expertise:

Health behavior, behavioral change, mixed methods, experimental study, qualitative study.

I confirm that I have read this submission and believe that I have an appropriate level of expertise to confirm that it is of an acceptable scientific standard.

# Reviewer report: Reviewer response for version 1

**DOI:** 10.5256/f1000research.159331.r350077

**Published:** 2024-12-26

**Authors:** Ramesh Kumar

**Affiliations:** 1 Department of Public Health, Health Services Academy, Islamabad, Pakistan

The study evaluates the "Men in Maternity" (MiM) intervention in Naypyitaw, Myanmar, to improve male involvement in maternal health care (MHC). Using a mixed-methods approach, the intervention significantly enhanced male partners' knowledge, attitudes, and practices, leading to higher institutional delivery (ID) rates. Challenges included cultural barriers, financial constraints, and personnel shortages.

 Strengths

1. **Innovative Intervention:** This paper effectively addresses a key gap by involving men, a novel approach for improving MHC outcomes in Myanmar.
2. **Robust Design:** The mixed-methods approach ensures a comprehensive evaluation of the intervention’s effectiveness, acceptability, and feasibility.
3. **Mixed Methods:** Significant improvements in knowledge and ID rates were complemented by qualitative findings that highlighted cultural shifts and positive stakeholder feedback.
4. **Policy Implications:** The study provides evidence-based recommendations that are adaptable to other low-resource settings

Weaknesses

1. **Limitations:** Financial constraints, cultural barriers, and limited trained personnel may hinder large-scale implementation.
2. **Time constrains:** The 6-month duration may not capture long-term sustainability or the full impact of the MiM program.
3. **Generalizability:** Findings are specific to Naypyitaw and may not represent other regions in Myanmar with different socio-cultural contexts.

This is an excellent paper; however, please include the study design in the methods section, provide a reference for the tool used, mention the ethical approvals or consent procedures (written/informed), outline the implications for practice, specify the outcome measures, and detail the tests performed in the study.

Is the work clearly and accurately presented and does it cite the current literature?

Yes

If applicable, is the statistical analysis and its interpretation appropriate?

Yes

Are all the source data underlying the results available to ensure full reproducibility?

Yes

Is the study design appropriate and is the work technically sound?

Yes

Are the conclusions drawn adequately supported by the results?

Yes

Are sufficient details of methods and analysis provided to allow replication by others?

Yes

Reviewer Expertise:

Related to public health

I confirm that I have read this submission and believe that I have an appropriate level of expertise to confirm that it is of an acceptable scientific standard.
